# Early-life adversity selectively impairs α2-GABA_A_ receptor expression in the mouse nucleus accumbens and influences the behavioral effects of cocaine

**DOI:** 10.1016/j.neuropharm.2018.08.021

**Published:** 2018-10

**Authors:** Scott J. Mitchell, Edward P. Maguire, Linda Cunningham, Benjamin G. Gunn, Matthias Linke, Ulrich Zechner, Claire I. Dixon, Sarah L. King, David N. Stephens, Jerome D. Swinny, Delia Belelli, Jeremy J. Lambert

**Affiliations:** a Division of Neuroscience, Medical Research Institute, Ninewells Hospital & Medical School, Dundee University, Dundee DD1 9SY, Scotland, United Kingdom; b Institute of Human Genetics, Mainz University, Medical Center, Mainz, Germany; c Institute for Biomedical & Biomolecular Sciences, School of Pharmacy & Biomedical Sciences, University of Portsmouth, Portsmouth PO1 2DT, United Kingdom; d School of Psychology, University of Sussex, Falmer, Brighton, BN 9QG, United Kingdom

**Keywords:** Early-life adversity, Early-life stress, GABA_A_ receptors, Nucleus accumbens, Cocaine

## Abstract

Haplotypes of the *Gabra2* gene encoding the α2-subunit of the GABA_A_ receptor (GABA_A_R) are associated with drug abuse, suggesting that α2-GABA_A_Rs may play an important role in the circuitry underlying drug misuse. The genetic association of *Gabra2* haplotypes with cocaine addiction appears to be evident primarily in individuals who had experienced childhood trauma. Given this association of childhood trauma, cocaine abuse and the *Gabra2* haplotypes, we have explored in a mouse model of early life adversity (ELA) whether such events influence the behavioral effects of cocaine and if, as suggested by the human studies, α2-GABA_A_Rs in the nucleus accumbens (NAc) are involved in these perturbed behaviors. In adult mice prior ELA caused a selective decrease of accumbal α2-subunit mRNA, resulting in a selective decrease in the number and size of the α2-subunit (but not the α1-subunit) immunoreactive clusters in NAc core medium spiny neurons (MSNs). Functionally, in adult MSNs ELA decreased the amplitude and frequency of GABA_A_R-mediated miniature inhibitory postsynaptic currents (mIPSCs), a profile similar to that of α2 “knock-out” (α2^−/−^) mice. Behaviourally, adult male ELA and α2^−/−^ mice exhibited an enhanced locomotor response to acute cocaine and blunted sensitisation upon repeated cocaine administration, when compared to their appropriate controls. Collectively, these findings reveal a neurobiological mechanism which may relate to the clinical observation that early trauma increases the risk for substance abuse disorder (SAD) in individuals harbouring haplotypic variations in the *Gabra2* gene.

## Highlights

• Early-life adversity (ELA) is a risk factor for adult psychopathology and drug abuse.
• *Gabra2* gene variations coupled with childhood trauma associate with cocaine abuse.
• ELA selectively impaired accumbal α2-GABA_A_R expression and function in adult mice.
• ELA and α2^−/−^ mice displayed similar abnormal behavioral responses to cocaine.
• The findings complement clinical associations of ELA, *Gabra2* gene and drug abuse.

## 1 Introduction

Drug addiction has both social and biological causes (Bierut, 2011, Enoch, 2012, Kalda and Zharkovsky, 2015). For example, studies with twins revealed that heritability accounted for ∼50% and 60–70% of the risk of an individual developing alcoholism and cocaine and opiate addiction respectively.(Goldman et al., 2005). Experience of childhood trauma predisposes to subsequent drug dependence (Delavari et al., 2016, Teicher et al., 2016), but this progression is markedly influenced by genetic makeup (Enoch, 2011, Enoch, 2012). Linkage and association studies identify variations in a region of chromosome 4 containing four GABA_A_R-subunit genes, (*Gabra2, Gabra4, Gabrb1, Gabrg1*), that confer an increased risk for developing substance abuse disorder (SAD; Edenberg et al., 2004). In support, we reported that haplotypes of the *Gabra2* gene encoding the GABA_A_R α2-subunit are associated with cocaine abuse in addicts (Dixon et al., 2010). Related variations of the haplotype are additionally linked to abuse of other drugs, suggesting that α2-GABA_A_Rs may play a key role in the circuitry underlying substance abuse (Covault et al., 2004, Enoch, 2011, Stephens et al., 2017). Importantly, the genetic association of *Gabra2* haplotypes with cocaine addiction, and other forms of drug abuse, was evident only in individuals with experience of childhood trauma (Enoch et al., 2010).

In mammalian brain GABA_A_Rs are the major inhibitory ionotropic receptors and are composed of five subunits drawn from a palette of 19 proteins (α1-6, β1-3, γ1-3, δ, ε, θ, ρ1-3), which underpins the expression of ∼20–30 physiologically and pharmacologically distinct GABA_A_R isoforms (Olsen and Sieghart, 2009, Rudolph and Möhler, 2014). These receptor subtypes are expressed in a brain- and neuronal-specific manner and occupy a distinct location within the neuron. Consequently, specific receptor subtypes mediate, or influence particular behaviors (Olsen and Sieghart, 2009, Rudolph and Möhler, 2014). The nucleus accumbens (NAc) is a region intimately associated with reward, addictions and an important locus for influencing stress-induced behaviors (Carlezon and Thomas, 2009, Russo et al., 2010, Scofield et al., 2016). We reported that medium spiny neurons (MSNs) of the neonatal mouse NAc express synaptic α2-GABA_A_Rs and that in the adult, accumbal α2-GABA_A_Rs influence the behavioral effects of cocaine (Dixon et al., 2010).

It is now evident that exposure to stressful events during sensitive developmental periods may produce long-lasting changes in the connectivity of the mesolimbic neurocircuitry (Peña et al., 2017). Given the association of childhood trauma, drug abuse and *Gabra2* haplotypes (Enoch et al., 2010), we have utilised immunohistochemistry, electrophysiology and behaviour to explore in a mouse model of early-life adversity (ELA) (Rice et al., 2008, Gunn et al., 2013) if such experiences influence the behavioral effects of cocaine in adults and as suggested by clinical studies, whether accumbal α2-GABA_A_Rs are implicated in these perturbed behaviors (Heitzeg et al., 2014).

We report that neonatal experience of ELA produced in adult mice a highly selective decrease in the expression and function of inhibitory synaptic α2-GABA_A_Rs of accumbal MSNs. Behaviourally, adult ELA mice presented with an enhanced locomotor effect to acute cocaine and blunted sensitisation to chronic administration of this psychostimulant, consistent with this early experience producing an enduring neural plasticity. Intriguingly, adult α2^−/−^ mice displayed a similar electrophysiological and behavioral phenotype. Collectively, these observations are consistent with impaired expression of α2-GABA_A_Rs being a factor in the altered effects of cocaine exhibited by ELA mice. An α2-subunit SNP consistently implicated in SAD appears to decrease expression of this protein (Lieberman et al., 2015). Therefore, our findings in this mouse model of early adversity appear to complement the clinical literature linking childhood trauma and genetic variations in the *Gabra2* gene with increased risk of cocaine abuse (Dixon et al., 2010, Enoch et al., 2010). Consequently, such mice may provide an insight into how genetic variations and the environment interact to perturb brain circuitry, thereby predisposing to substance abuse, but also to influence other psychological disorders such as anxiety and depression. (Low et al., 2000, Dixon et al., 2008, Benham et al., 2017).

## 2 Materials and methods

### 2.1 Animals

Colonies of mice were maintained at the University of Dundee. All experimentation was conducted according to the ARRIVE guidelines on animal research (https://www.nc3rs.org.uk/arrive-guidelines). All procedures were performed in accordance with the Animals (Scientific Procedures) Act of 1986, after review by the University of Dundee Ethical Review Committee and under the licenses of Dr. Belelli (60/4005 & 70/8161) and Prof. Hales (70/8404). All mice employed in this study, (wild type (WT), α2^−/−^, α1^−/−^, α1H101R and α2H101R), were generated on a mixed C57BL/6J-129SvEv background as previously described (McKernan et al., 2000, Sur et al., 2001, Dixon et al., 2008). Mice were group housed, given free access to water and standard rodent chow (Special Diet Service U.K.), maintained on a 12 h alternating light-dark regimen with lights on at 7–7:30 a.m. The temperature and the humidity were controlled at 21 ± 2 °C and 50 ± 5% respectively. All experiments (behaviour, electrophysiology, immunohistochemistry, qPCR) were conducted on mice/tissue obtained from the first two generations of WT and mutant breeding pairs, the latter derived from the corresponding heterozygous mice, bred at Dundee University. Tissue required for immunohistochemistry (Portsmouth University) and for qPCR studies (Mainz University) was prepared in Dundee and then shipped as required.

### 2.2 The early-life adversity (ELA) paradigm

We previously described the ELA paradigm of fragmented maternal care (Gunn et al., 2013), which was based on the publication by Rice et al. (2008), with minor modifications. Timed, mated, pregnant, female mice were individually housed and monitored every 12 h for the birth of pups, with the day of birth designated as P0. All dams and their pups were left undisturbed until P2. If required, on day P2 the litters were reduced to 6–8 pups of either gender. Initially, all dams and their offspring were housed in a transparent cage, furnished with standard sawdust on the cage floor (650 ml) and were provided with a square (5 × 5 cm) of cotton nesting material (Nestlet DBM West Lothian, Scotland), which the dam subsequently shredded to form a nest.

At P2 the control dam and her pups were transferred to another identical control cage, whereas for the ELA protocol the dam and pups were transferred to a similar cage to the control cage, which however, contained reduced nesting material (2/3 of the cotton square) and a fine gauge (50 mm) steel mesh platform floor, raised ∼ 2.5 cm above the sawdust covered cage floor. Both control and ELA mice were left undisturbed until P9, when all pups with their dam were transferred to a “control cage”, which lacked the raised floor and was furnished with standard bedding and nesting material. All control and ELA pups remained with their dam under normal husbandry conditions until weaning at P21 when the cages were again changed. The behaviour of WT control; WT ELA and α2^−/−^ dams was determined by analysis of video camera recordings; 3 × 30 min sessions a day during the light (08:30; 15:00) and dark (22:00) phases on days 3–8 of the life of the offspring (Gunn et al., 2013). The number of sorties of the dam from the nest (*i.e*. the number of times the dam leaves the nest) was determined for each session, was then totalled for the day and then totalled across the period of investigation (p 3–8) – Gunn et al., 2013. Statistical comparisons of the number of sorties *per* hour for control WT *vs* ELA WT and for control WT *vs* α2^−/−^ mice were made using the unpaired Student's *t-*test.

All electrophysiology, immunohistochemistry, molecular biology and cocaine behavioral studies were performed on male mice. As regards female mice, during their ovarian cycle the levels of certain neurosteroids, that act as endogenous positive allosteric modulators of the GABA_A_R, are known to fluctuate substantially (Belelli and Lambert, 2005). Given that such steroids influence the expression of the GABA_A_R α2-subunit (Reddy et al., 2017) our initial focus was limited to male mice. However, given that female rodents are reportedly more resilient than their male counterparts to the ELA paradigm (Walker et al., 2017), future comparative gender experiments are warranted.

### 2.3 Electrophysiology

WT control, WT ELA, α2^−/−^, α2H101R^+/+^, α1H101R^+/+^ mature male mice (>2 months) were killed by cervical dislocation in accordance with Schedule 1 of the U.K. Government Animals (Scientific Procedures) Act of 1986. Coronal slices incorporating the NAc (300 μM thick) were prepared as we previously described (Dixon et al., 2010, Maguire et al., 2014). During the slice preparation, the tissue was sustained in oxygenated ice-cold solution containing the following (mM): 140 K gluconate, 15 Na gluconate, 4 NaCl, 10 HEPES and 0.2 EGTA, pH 7.2, 310–320 mOsm. Such slices were then maintained in a holding chamber (room temperature) containing an oxygenated extracellular solution (ECS) composed of (mM): 126 NaCl, 26 NaHCO3, 2.95 KCl, 1.25 NaH2PO4, 2 MgCl_2_, 2 CaCl2, 10 glucose, (305–310 mOsm, pH 7.4). The slices were left for > 1hr. before making recordings. Recording electrodes were prepared from thick-walled borosilicate glass (Garner Glass Company), fabricated to have open tip resistances of 3 – 5MΩ, when filled with intracellular solution. Whole-cell, voltage-clamp recordings were made from visually identified MSNs of the NAc core (using an Olympus BX50WI microscope) at 35 °C, with the majority of recordings made at a holding potential (Vh) of −60 mV, apart from those experiments investigating the glutamatergic “tonic” current where the holding potential was +40 mV (see below). In all cases, recordings were discarded if the series resistance changed (20% tolerance) during the course of the experiment.

For recording GABA_A_R-mediated phasic (mIPSCs) and tonic currents the patch pipette was filled with an intracellular solution containing (mM): 135 CsCl, 10 HEPES, 10 EGTA, 1 CaCl_2_, 2 MgCl_2_, 2 Mg-ATP, 5 QX-314 (pH 7.2–7.3 with CsOH, 300–308 mOsm). The slice was perfused with an ECS containing 1 μM strychnine, 2 mM kynurenic acid, and 0.5 μM tetrodotoxin (TTX). To investigate the effect of ELA on ionotropic glutamate receptor-mediated phasic events (excitatory postsynaptic currents – EPSCs) and on tonic currents of NAc core MSNs, we utilised the recording conditions previously described (Gunn et al., 2013). The ECS was similar to that used for recording GABA_A_R-mediated currents in the current study, with the exceptions that it additionally contained bicuculline (30 μM), with kynurenic acid omitted, and a reduced MgCl_2_ (0.5 mM) concentration, the latter, in part to increase the magnitude of the NMDAR-mediated tonic current (see below), but additionally to enhance the mEPSC frequency. However, under these ECS conditions initial experiments revealed a low phasic event frequency, consequently impairing meaningful quantification of the mEPSC parameters. Therefore we additionally excluded TTX to now record sEPSCs. Note we had previously found in the PVN that prior ELA increased the frequency of both sEPSCs and mEPSCs (Gunn et al., 2013). The ICS was composed of in mM: 135 CH_3_O_3_SCs, 8 CsCl, 10 HEPES, 10 EGTA, 1 MgCl_2_, 1 CaCl_2_, 300–310 mOsm, pH 7.2–7.3 with CsOH (Gunn et al., 2013). The sEPSCs were recorded at a Vh of −60 mV.

The inhibitory tonic current mediated by GABA primarily activating extrasynaptic α4βδ-GABA_A_Rs was determined by quantifying the change in the holding current (Vh = −60 mV) induced by the GABA_A_R antagonist bicuculline (30 μM) - see previous publications (Belelli et al., 2005, Maguire et al., 2014). Preliminary experiments established that core MSNs additionally exhibited a tonic current, primarily mediated by extrasynaptic NMDA receptors. To investigate such tonic currents, recordings were made in the presence of a GABA_A_R antagonist (gabazine; 10 μM) at a Vh of +40 mV and in a low MgCl_2_ (0.1 mM) - containing ECS, conditions to facilitate the unblocking of the NMDA receptor associated ion channel by Mg^2+^. The pipette solution was composed of: 135 CH_3_O_3_SCs, 1 EGTA, 10 HEPES, 5 TEA-Cl, 1 MgCl_2_, 0.5 Na GTP, 2 Mg-ATP, 5 Tris phosphocreatine (280–290 mOsm pH 7.2–7.3 with CsOH). The change in holding current in response to either kynurenic acid (2 mM), or APV (50 μM) was determined as previously described (Gunn et al., 2013).

### 2.4 Electrophysiology data and statistical analysis

Currents were filtered at 2 kHz using an eight-pole low-pass Bessel filter and recorded *via* an A/D converter (NIDAQmax: National Instruments) at a sampling rate of 10 kHz stored on the computer hard drive for subsequent offline analysis using the Strathclyde Electrophysiology Software, Electrophysiology Data Recorder/Whole-Cell Analysis Program (WinEDR/WinWCP; Dr. J. Dempster, University of Stratchclyde). The mIPSCs and sEPSCs were detected using an automated low amplitude (−4 pA, rise time duration of 1.0 ms) threshold detection algorithm and visually inspected for validity. Accepted events (at least 50 for each recording condition and each with a rise time ≤ 1 ms) were analysed with respect to their peak amplitude, rise time (10–90%) and their decay time course. A minimum of 50 events were also digitally averaged by alignment at the midpoint of the rising phase. For mIPSCs a least-squares minimization algorithm was used to determine the decay time constant. The decay phase of such averaged events was fitted (98–10% of the peak amplitude) by either:

a mono-exponential: [y(t) = A.e(-t/τ)], or

a bi-exponential function: [y(t) = Afast.e(−t/τfast) + Aslow.e(−t/τslow)],

where: t = time, A = amplitude, τ = the decay time constant. Analysis of the SD of residuals and use of the F-test to compare goodness of fit revealed that the averaged event decay was always best fit with the sum of 2 exponential components. Thus, a weighted decay time constant (τw) was also calculated:

τw = τ1P1 + τ2P2

τ1 and τ2 = the decay time constants of the first and second exponential functions, P1 and P2 = the proportion of the synaptic current decay described by each component.

To determine the mIPSC, or sEPSC frequency, events were automatically detected using the EDR program on the basis of their rate of rise (30–50 pA ms^−1^) and subsequently manually scrutinized to exclude spurious noise and include events that had failed to meet the trigger specifications. All data are presented as the arithmetic mean ± SEM. When data are presented normalised, the mean value was calculated by averaging the normalised change for each cell following drug application. The statistical significance of mean data was assessed using two-tailed paired and two-tailed unpaired Student's *t*-tests, or one- or two-way RM ANOVA followed by *post-hoc* Tukey test as appropriate, using the GraphPad Prism 7. Statistical significance was set at p < 0.05. The nonparametric Kolmogorov-Smirnoff test was used to compare cumulative probability distributions. For a stringent comparison, statistical significance was set at p < 0.01 for the Kolmogorov-Smirnoff test.

For GABA- and glutamate-mediated tonic currents the holding current was sampled every 102.4 ms for a 1 min period. At a sampling rate of 10 kHz 1024 baseline points for each 102.4 ms provided one data point. Epochs containing phasic events, or an unstable holding current were excluded. To validate that the drug-induced changes in holding current were not due to temporal “drift” two discrete 1 min sections of the recording were analysed for control (C1 and C2) and a 1 min section analysed once the receptor antagonist effect had reached equilibrium (D). The mean DC values for each control epoch were pooled and the SD determined. The drug effect was considered genuine if the absolute change in the holding current (D – C2) was greater than twice the SD associated with the DC measurements for the control period (Gunn et al., 2013).

### 2.5 Immunohistochemistry

The quantitative immunohistochemical data are derived from five adult (8–10 weeks old) C57BL6-129SvEv male mice, that had previously experienced the control, or the ELA cage condition (Gunn et al., 2013, Maguire et al., 2014). Qualitative data are derived from four adult (8–10 weeks old) C57BL6 male mice, that had previously experienced the control condition. Briefly, anaesthetised animals were transcardially perfused with 0.9% saline solution for 3 min, followed by 12 min fixation with a fixative consisting of 1% paraformaldehyde, 15% v/v saturated picric acid, in 0.1 M phosphate buffer (PB), pH 7.4. The brains were post-fixed in the same fixative solution overnight at 4 °C, then transferred to phosphate buffer containing 0.05% sodium azide and shipped from Dundee to Portsmouth.

Coronal sections (50 μm thick) were prepared on a Vibratome. For antigen retrieval, the tissue sections were incubated at 37 °C for 10 min in 0.1 M PB, followed by 15 min in 0.2 M HCl containing 1 mg/ml pepsin (Sigma, UK), after which they were washed thoroughly in Tris-buffered saline containing 0.3% Triton-X100 (TBS-Tx) for 30 min. Non-specific binding of secondary antibodies was blocked by incubating sections with 20% normal horse serum for 2 h at room temperature. The tissue sections were incubated with combinations of the following primary antibodies: goat anti DARPP-32, (Santa Cruz, # 8483), 1:250; guinea pig anti GABA_A_R α1 subunit (Synaptic Systems, # 224,205), 1:1000; rabbit anti GABA_A_R α2 subunit (raised against amino acids 416–424 of the C-terminus, a generous gift of Werner Sieghart), diluted in TBS-Tx, for 24- hr. at 4 °C. After washing with TBS-Tx, sections were incubated in a mixture of appropriate secondary antibodies conjugated with either Alexa Fluor 488 (Invitrogen, Eugene, OR), indocarbocyanine (Cy3; Jackson ImmunoResearch), and indodicarbocyanine (Cy5; Jackson ImmunoResearch) for 2 h at room temperature. Sections were washed in TBS-Tx and mounted in Vectashield (Vector Laboratories, Burlingame, CA). The specificities of the GABA_A_R α1 and α2 subunit antisera used in this study were confirmed in NAc tissue slices from GABA_A_R α1^−/−^ and α2^−/−^ mice respectively. To confirm the absence of cross reactivity between IgGs in double and triple immunolabelling experiments, some sections were processed through the same immunocytochemical sequence, except that only an individual primary antibody was applied with the full complement of secondary antibodies.

### 2.6 Image acquisition

Sections were examined with a confocal laser-scanning microscope (LSM710; Zeiss, Oberkochen, Germany) using either a Plan Apochromatic 63× DIC oil objective (NA1.4), or a Plan Apochromatic 100× DIC oil objective (NA1.46). Z-stacks were used for routine evaluation of the labelling. All images presented represent a single optical section. These images were acquired using sequential acquisition of the different channels to avoid cross-talk between fluorophores, with the pinholes adjusted to one airy unit. Images were processed with the software Zen2009 Light Edition (Zeiss, Oberkochen, Germany) and exported into Adobe Photoshop. Only brightness and contrast were adjusted for the whole frame, and no part of a frame was enhanced, or modified in any way.

### 2.7 Quantification of GABA_A_R α1-and α2-subunit immunoreactivity in the NAc and statistical analysis

We have previously described the quantitative methods (Gunn et al., 2013). All sections were processed and imaged under identical conditions and analyses were performed blind. GABA_A_R α1-and α2-subunit immunoreactivity presented as distinct, individual clusters. We, therefore, quantified the density of immunoreactive clusters, expressed both as the number of individual clusters *per* 10 μm^2^ and as the average area of clusters, expressed as μm^2^. In neurons, GABA_A_Rs cluster together predominantly at synapses, but may also cluster on extrasynaptic membrane micro-domains. Therefore analysis of such cluster immunoreactivity represents expression in such sub-cellular domains. However, it is important to note that the antibody recognises particular subunits and cannot unconditionally be equated directly to receptor expression. Taking cognisance of that limitation, a decrease in the density of such clusters could equate to fewer individual receptors distributed across the area of an individual neuron, whereas a decrease in the cluster area may reflect a reduced number of receptors distributed within specific sub-compartments, such as synapses. However, confirmation would ultimately require ultra-structure analyses employing immunohistochemistry at the transmission electron microscope level.

The imaging and quantification were performed as follows: within a tissue section, 3 fields of view (FOV) were randomly selected within the NAc core. Z-stacks consisting of three optical sections spaced 5 μm apart in the Z plane were acquired for each FOV. The dimensions of each optical section were 85 μm × 85 μm x 1 μm in the X-Y-Z planes. Within an optical section, the number of α-GABA_A_R subunit clusters was manually counted and the average area of individual clusters measured, using ImageJ software. A value for each FOV was obtained by computing the average from the optical sections contained within a FOV, and these were averaged for an individual tissue section. This was repeated in ∼4–5 tissue sections *per* mouse. The mean ± SEM cluster density, or area, from 5 mice was then determined.

An unpaired Student's *t*-test was used to determine statistically significant differences (*p* < 0.01) between WT control and WT ELA mouse tissue. In a subset of experiments, we also quantified the density of individual GABA_A_R α-subunit immunoreactive clusters which contacted VGAT-immunoreactive puncta.

### 2.8 Quantitative RT-PCR analysis

WT control and WT ELA coronal slices incorporating the NAc were prepared as described above for the electrophysiological studies, with the exception that the slices were 800 μM thick (Dixon et al., 2010, Maguire et al., 2014). Accumbal “punches” were then made and the tissue manually trimmed using a scalpel and dissecting microscope. The accumbal “punches” of 3 control and 3 ELA mice were subsequently weighed and snap-frozen in liquid nitrogen prior to being shipped on dry ice to Mainz University for RT-qPCR studies. Total RNA from punches was isolated with RNAeasy Mini Kit (Qiagen, Hilden, Germany), following the manufacturer's instructions. RNA quantification was performed by measurements with the Qubit^®^ RNA BR Assay Kit (Life Technologies, Darmstadt, Germany) according to manufacturer's instructions. The cDNA was synthesized from 50 ng total RNA by reverse transcription using oligodT and random hexamer primers with the PrimeScript™ RT Reagent Kit (TaKaRa Bio, Shiga, Japan) according to the manufacturer's instructions. The cDNA samples were then diluted 1:8, and 2 μl of the diluted cDNA was used for quantitative PCR (qPCR) of the GABA_A_R subunit genes (Table 1) with SYBR Premix Ex Taq II (Tli RNaseH Plus), ROX plus (TaKaRa Bio, Shiga, Japan) on a StepOnePlus Real-Time PCR System (Life Technologies, Darmstadt, Germany). Data was first explored with LinRegPCR (Ramakers et al., 2003) for calculating PCR efficiency and subsequently analysed using the REST software with PCR efficiency correction (Pfaffl et al., 2002).

**Table 1:** Genes and primers for the RT-qPCR analysis.

Gene	Sequence Forward Primer (5′-3′)	Sequence Reverse Primer (5′-3′)
*Gabra1*	CACCATGAGGTTGACCGTGA	CTACAACCACTGAACGGGCT
*Gabra2*	ACTAGCTGTTCAGCTTTGGCA	ATGTTAGCCAGCACCAACCT
*Gabra4*	ACGAGAAATTGTGCCCGGAA	CACTTCTGTAACAGGACCCCC
*Gabrd*	GGCGCCAGGGCAATGAAT	AAGTTTCGGGCATAGCCCTC

The relative expression ratio of a target gene was computed, based on its RT-qPCR efficiencies (*E*) and the threshold cycle difference (ΔCt) for 3 ELA samples *versus* 3 WT controls. With this approach the target gene expression is normalised to the expression of non-regulated reference genes (in this study *Gapdh* and *Ub2q1*), based on the following equation:

Ratio = (*E*_*target*_)^ΔCt^target (WT control – ELA sample)/(*E*_*ref*_)^ΔCt^ref (WT control – ELA sample).

Statistical significance was explored using the Pair Wise Fixed Reallocation Randomisation Test^©^ which forms part of the REST software. This test assesses the probability of the alternate hypothesis that the difference between the sample and control groups is due only to chance (P(H1). To devise a strong randomisation test, we employed the following randomisation scenario: “if any perceived variation between samples and controls is due only to chance, then we could randomly swap values between the 2 groups and not observe any greater difference than the difference we observe between the initial groups.” The hypothesis test performs 10,000 random reallocations of samples and controls between the groups, and counts the number of times the relative expression on the randomly assigned group is greater than the sample data (REST, 2009 Software User Guide 12/2009).

### 2.9 Cocaine behavioral studies

The locomotor activity of adult (2–4 months of age) male mice was assessed using a system developed at the University of Sussex. Sixteen black Perspex, circular runways (internal diameter, 11 cm; external diameter, 25 cm; height, 25 cm) were set above a translucent platform. Illumination of the runways was achieved by 2 fluorescent tubes (T4, 30 W) positioned above a translucent Perspex sheet suspended 20 cm above the runways. Mice were videoed from below through a translucent Perspex floor by a camera (Fire-i; UniBrain, Scorpion Vision Software, Hampshire, UK), that detected the moving shadow of the subject. Images were digitized, recorded and locomotor activity determined using Sussex University software written in Matlab (version 2007a, The MathWorks, Cambridge, UK). The overall distance travelled was measured in meters (m). Experiments were conducted between 08:00 a.m. and 13:00 p.m. Before experimentation, all mice were habituated to the circular runways in two, once-daily, sessions. Following habituation for all experiments mice were initially placed in the runways for 30 min, then returned to the home cage for 5 min (during which the apparatus was cleaned). Immediately following either a single *i.p.* injection of saline, or of cocaine (10 mg/kg), mice (WT control, WT ELA and α2^−/−^ control) were then again placed in the runways for a further 60 min. The first 15 min following injection was used for analysis as the cocaine-induced increase of locomotor activity decreased after this time. To investigate sensitisation, all groups received repeated, daily injections of saline, or cocaine (10 mg/kg) for 10 sessions, across 12 days (*i.e*. 5 days of treatment, then 2 day without treatment, followed by a further 5 days of treatment). In a separate cohort of mice doses of 0, 10 and 20 mg/kg were tested to confirm 10 mg/kg to be a submaximal effective dose for WT control, WT ELA and α2^−/−^ mice. In both the repeated cocaine/vehicle administration paradigm and the dose-response study, the groups and treatments were randomised, with the experimenter blinded to the variables of drug treatment, or of mouse group.

### 2.10 Statistical analysis of behaviour

Two-tailed paired and two-tailed unpaired Student's t-tests were performed for comparing the means between two dependent and independent groups respectively. For more than two groups an analysis of variance (ANOVA), or a repeated measures ANOVA (RM ANOVA), one- or two-way, were used as appropriate. For all tests, statistical differences were assumed to be significant when p < 0.05. When a test was significant further analysis was performed using Tukey *post-hoc* analysis, ANOVA and individual between- or within-genotype comparisons by *t*-test.

### 2.11 Reagents and drugs

Bicuculline methobromide (ENZO Life Sciences), strychnine hydrochloride (Sigma-Aldrich) kynurenic acid, D-APV (Abcam), gabazine (HelloBio) and TTX (Tocris Bioscience), were prepared as aqueous stock solutions and subsequently diluted in ECS to the desired final concentration. Zolpidem (Tocris Bioscience) was prepared as a concentrated (x 1000) stock solution in DMSO and then diluted in ECS to the required concentration. The final DMSO concentration (0.1%v/v) had no effect on mIPSCs. Cocaine hydrochloride (Sigma) was prepared in a 0.9% saline solution and used on the day of preparation.

## 3 Results

### 3.1 In common with deletion of the GABA_A_R α2-subunit, prior ELA selectively decreases phasic inhibition in nucleus accumbens core MSNs

**Fig. 1:**
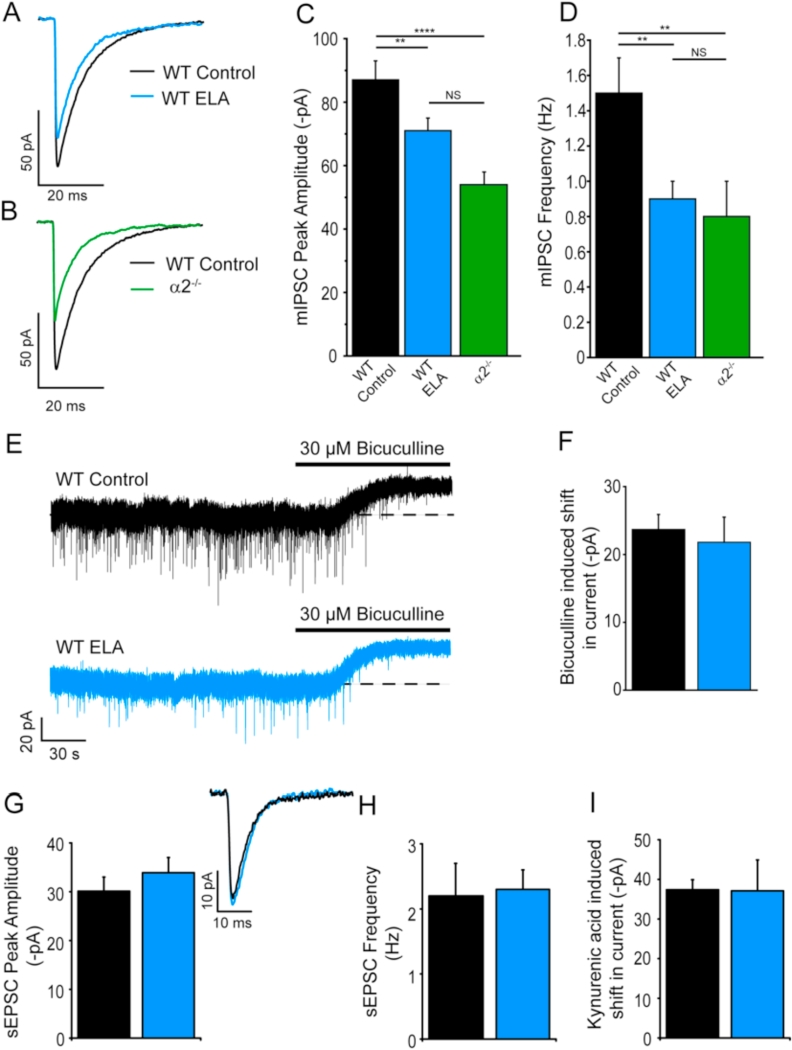
In common with deletion of the α2-subunit, prior experience of ELA produced a selective decrease of phasic GABA_A_R-mediated inhibition in adult mouse NAc. MSNs. Note for all traces and bar charts the control, ELA and α2^−/−^ condition are black, blue and green respectively. **A, B)** Illustrated are superimposed, representative, averaged GABA_A_R-mediated mIPSCs, recorded from accumbal core MSNs, demonstrating the effect of **A)** prior ELA (WT ELA), and **B)** the α2 subunit deletion (α2^−/−^) upon the mIPSC amplitude compared to WT controls. A decrease in the mIPSC peak amplitude is evident for both conditions. **(C, D)**. Bar charts (mean ± S.E.M) comparing against WT controls the impact of prior ELA and of deleting the GABA_A_R α2-subunit upon **(C)** the peak amplitude and **(D)** the frequency of GABA_A_R-mediated mIPSCs. Prior ELA (n = 25), or the deletion of the α2-subunit (n = 22), significantly decreased both the mIPSC peak amplitude and their frequency compared to WT (n = 51) –compare also the traces in panel **E** where the ELA-induced decrease in mIPSC frequency is clearly evident. The statistical significance was determined by a one-way RM ANOVA followed by a Tukey *post-hoc* test (for panels **C,** & **D**) - **p < 0.01; ****p < 0.0001, NS = non-significant. **(E)** Illustrated are representative traces of MSN whole-cell current recordings obtained from a WT control and a WT ELA mouse before and after bicuculline application. For both conditions, the GABA_A_R antagonist bicuculline (30 μM) produced a similar outward current and an associated decrease in the membrane noise. The broken lines indicate the mean holding current prior to bicuculline application. Note with this relatively slow time scale the mIPSCs appear as relatively brief downward deflections. Comparison of these traces illustrates the ELA-induced decrease in mIPSC frequency. **(F)** A bar chart (mean ± s.e.m) demonstrating that the outward current produced by bicuculline (30 μM) is not influenced by prior experience of ELA (WT control n = 32; WT ELA n = 10; p = 0.2465, unpaired *t*-test *versus* WT control). **(G**–**I)** Bar charts (mean ± s.e.m.) demonstrating that prior experience of ELA has no effect on the **G)** peak amplitude, or **H**) frequency of sEPSCs, (n = 7–9; unpaired Student's t-test; p = 0.41 and 0.664 respectively), or **I)** on the magnitude of the excitatory tonic current (see Methods) revealed by 2 mM kynurenic acid (control and ELA n = 6; unpaired Student's t-test p = 0.97). Additionally, Panel **G** illustrates representative superimposed WT control (black) and WT ELA (grey) averaged sEPSCs. (For interpretation of the references to colour in this figure legend, the reader is referred to the web version of this article.)

In agreement with our previous study and that of others (Rice et al., 2008, Gunn et al., 2013) the reduced bedding paradigm significantly increased the number of sorties of the dam from the nest (WT control = 29.6 ± 3.03/hr. n = 9; WT ELA = 50.5 ± 3.7/hr. n = 7; t_(14)_ = 4.437; p = 0.0006). Having confirmed a disrupted mother-pup interaction, we utilised the whole-cell voltage-clamp technique to investigate the impact of ELA on inhibitory and excitatory neurotransmission in the NAc core MSNs of adult (>2 months old) mice. Prior experience of ELA in comparison to WT control conditions produced a significantly reduced mIPSC amplitude (ELA = −68 ± 4 pA, n = 25; control = −87 ± 4 pA, n = 51, *post-hoc* Tukey test p = 0.0080, F(2,95) = 15.94, p = 0.001, one-way ANOVA) and frequency (ELA = 0.9 ± 0.1 Hz, n = 25; control = 1.5 ± 0.1 Hz, n = 51, *post-hoc* Tukey test p = 0.0024, F(2,95) = 8.31, p = 0.0005, one-way ANOVA), but with no change to their kinetics, (Fig. 1A,C,D).

We reported that, in addition to phasic inhibition mediated by synaptic GABA_A_Rs, adult NAc. core MSNs express extrasynaptic GABA_A_Rs composed of α4, β and δ subunits, that mediate a tonic inhibitory current, due to their activation by ambient concentrations of GABA (Maguire et al., 2014, Stephens et al., 2017). This tonic current, revealed by the application of bicuculline (30 μM), was similar (t_(40)_ = 1.176, p = 0.2465, unpaired *t-*test) for MSNs derived from control (24 ± 2 pA; n = 32) and ELA mice (22 ± 4 pA; n = 10) – (Fig. 1E and F). Hence, prior ELA selectively compromised GABA-ergic phasic, but not tonic inhibition.

We observed that ELA produced an increase in glutamatergic drive, to the CRF-releasing neurons of the paraventricular nucleus (PVN), manifest as an increase in the frequency of both mEPSCs and sEPSCs, with, in contrast to the NAc core, relatively little effect on GABAergic inhibition (Gunn et al., 2013). In preliminary control recordings the frequency of NAc mEPSCs was relatively low, hampering meaningful analysis of EPSC properties (See Methods Section 2.3). Therefore, we investigated the influence of ELA upon sEPSCs, mediated by synaptic ionotropic AMPA receptors. In contrast to the PVN, ELA had no significant effect upon the sEPSC frequency, or amplitude (t_(18)_ = 0.4421, p = 0.6637 and t_(14)_ = 0.8553, p = 0.4068 respectively, unpaired *t*-test) – Fig. 1G and H. For PVN neurons, ELA additionally caused a large increase in an excitatory tonic conductance mediated primarily by extrasynaptic NMDA receptors (Gunn et al., 2013). Here, at a holding potential of +40 mV, in a low magnesium ECS (see Methods), for all MSNs tested, the non-selective ionotropic glutamate receptor antagonist kynurenic acid (2 mM) induced an inward current, (37 ± 3 pA; n = 6), primarily mediated by NMDA receptors (a similar current was produced by the NMDA receptor antagonist APV - 50 μM – not shown). In contrast to the PVN, ELA had no effect on the magnitude of this tonic excitatory current revealed by kynurenic acid (32 ± 1 pA; n = 7) *versus* WT control (t_(10)_ = 0.03063, p = 0.9762, unpaired *t*-test) - Fig. 1I. In summary, in NAc core MSNs, prior experience of ELA selectively impaired phasic inhibition, with no effect on tonic inhibition, or phasic and tonic excitatory transmission. Future studies will investigate sIPSCs to determine whether network driven GABA release is influenced by prior ELA.

Regarding the ELA-induced impairment of phasic inhibition, (mIPSCs) we had previously shown that synaptic α2-GABA_A_Rs are expressed in NAc core MSNs of relatively young (p17-24) neonatal mice (Dixon et al., 2010). Given this finding and the association of ELA and *Gabra2* haplotypes (see Introduction) we next investigated in MSNs from adult mice the impact of genetically deleting the α2 subunit (α2^−/−^ mouse) on mIPSCs. A statistical analysis revealed an effect of mouse group upon mIPSC peak amplitude (F(2,95) = 15.94, p = 0.0001, one-way ANOVA) and frequency (F(2,95) = 8.31, p = 0.0005, one-way ANOVA). In common with ELA, the mIPSCs recorded from the MSNs of the α2^−/−^ adult mouse exhibited a reduced amplitude (−53 ± 3 pA, n = 22, p = 0.0001 *post-hoc* Tukey test) and frequency of occurrence (0.9 ± 0.2 Hz, n = 22, p = 0.0058 *post-hoc* Tukey test) compared to WT – Fig. 1 B,C,D.

To further investigate whether the impairment of phasic inhibition induced by prior ELA results from a selective decreased expression of the α2-subunit we now employed immunohistochemistry to determine the synaptic GABA_A_R isoform(s) that mediate phasic inhibition in the NAc core MSNs of adult control mice, with a focus on receptors incorporating the α2-and α1-GABA_A_R subunits, considered to be the predominant synaptic isoforms (Hortnagel et al., 2013).

### 3.2 GABA_A_Rs incorporating the α1-and α2-subunit are expressed on NAc core MSNs and mediate synaptic inhibition

**Fig. 2:**
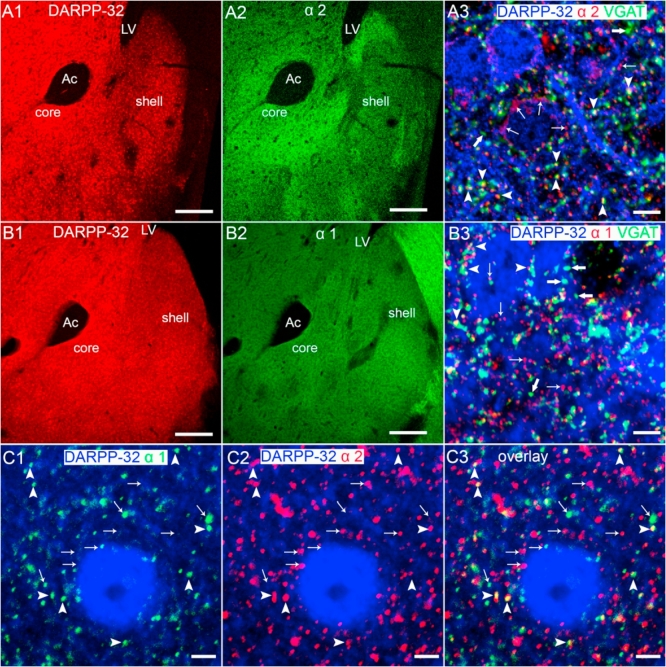
The immunolocalisation of the GABA_A_R α1 and the α2 subunit in the mouse nucleus accumbens core. (**A1, B1**) Illustrates the immunoreactivity for DARPP-32, a phospho-protein located selectively in dopamine receptor expressing neurons, which in the ventral striatum delineates the distribution of medium spiny neurons (MSNs). (**A2**) illustrates that immunoreactivity for the α2-subunit is expressed throughout the ventral striatum and closely follows the distribution of (**A1**) DARPP-32 immunolabelling, indicating expression in MSNs. Note the enrichment of the α2-subunit immunoreactivity within the core sub-region of the NAc compared to the shell. (**A3**) shows the association between the immunoreactivity for the GABA_A_R α2-subunit and the GABAergic axon terminal marker protein vesicular GABA transporter (VGAT) on DARPP-32 immunopositive profiles, which suggests a predominantly synaptic location for this subunit. Numerous α2-subunit immunoreactive clusters are contacted by VGAT immunopositive puncta (arrowheads). However, some α2-subunit immunoreactive clusters are not associated with VGAT puncta (thin arrows), whilst some VGAT puncta are not associated with α2-subunit immunoreactive clusters (thick arrows). (**B2**) illustrates the immunoreactivity for the GABA_A_R α1-subunit. In contrast to the α2-subunit staining, note the relatively even distribution of α1-subunit immunoreactivity between the core and the shell sub-regions. (**B3**) shows the association between immunoreactivity for the GABA_A_R α1-subunit and VGAT, on DARPP-32 immunopositive profiles, which suggests a predominantly synaptic location for this subunit. Numerous α1-subunit immunoreactive clusters are contacted by VGAT immunopositive puncta (arrowheads). However, some α1-subunit immunoreactive clusters are not associated with VGAT puncta (thin arrows), whilst some VGAT puncta are not associated with α1-subunit immunoreactive clusters (thick arrows). (**C**) Clusters immunoreactive for (**C1**) the GABA_A_R α1-subunit and (**C2**) the GABA_A_R α2-subunit are **(C3)** co-localised (arrowheads) on DARPP-32-immunopositive somata and dendrites. The thin arrows indicate individual clusters, which are not co-localised. Ac = anterior commissure; LV = lateral ventricle. Scale bars: A1,2; B1,2 = 200 μm; A3, B3 = 5 μm; C1-3 = 2 μm.

The distribution of MSNs within the NAc was revealed by immunoreactivity for DARPP-32 (Fig. 2A1, B1), a cytosolic protein, exclusively expressed by dopamine receptor-expressing neurons (Walaas and Greengard, 1984). In adult MSNs, immunoreactivity for the α2-subunit was clearly evident and was closely associated with that of DARPP-32, indicating a predominant localisation within the MSNs (Fig. 2A1,2). The specificity of NAc staining by the α2-subunit antibody was confirmed by immunohistochemistry of tissue derived from α2^−/−^ mice (not shown). There was a notable gradient of expression between the core and shell sub-regions of the NAc, with greater levels of the α2-subunit signal apparent in the former (Fig. 2A2). At greater resolution, the immunoreactivity pattern of the α2-subunit within the core appeared as distinct clusters, which were often closely opposed to puncta immunopositive for the vesicular GABA transporter (VGAT), a protein enriched with GABAergic axon terminals, suggesting an enrichment of α2-GABA_A_Rs within GABAergic synaptic junctions (Fig. 2A3). However, some of the α2-subunit immunopositive clusters were not associated with those immunopositive for VGAT, whilst some VGAT immunopositive clusters were not associated with α2-subunit immunopositive clusters. Quantification of the density of α2-subunit immunoreactive clusters contacted by VGAT immunopositive puncta, compared to the total density of α2-subunit immunoreactive clusters, revealed that ∼83% of α2-subunit immunoreactive clusters are located in proximity to putative GABA-ergic axon terminals (mean ± SEM; total α2-subunit density, 4.6 ± 0.03 clusters *per* 10 μm^2^ *vs* α2-subunit-VGAT density, 3.8 ± 0.03 clusters *per* 10 μm^2^, n = 3 mice). Those clusters of α2-subunit staining not co-localised with VGAT staining may represent populations of extrasynaptic α2-GABA_A_Rs.

For comparison, we investigated the staining pattern for the GABA_A_R α1-subunit. The antibody specificity was confirmed using NAc slices obtained from α1^−/−^ mice (not shown). In contrast to the α2-subunit, immunoreactivity for the α1-subunit appeared evenly distributed within the core and shell sub-regions of the NAc (Fig. 2B1,2). In common with α2-subunit staining, at high resolution, in the core α1-subunit immunoreactivity also presented as individual clusters located on DARPP-32-immunopositive somata and dendrites, with the majority of clusters closely opposed to puncta immunopositive for VGAT (Fig. 2B3; arrowheads). Although most α1-subunit immunoreactive clusters were contacted by VGAT immunoreactivity, some isolated α1-subunit immunoreactive clusters were not (Fig. 2B3; thin arrows), suggesting the expression of a population of extrasynaptic α1-GABA_A_Rs. Additionally, some VGAT immunopositive puncta were not associated with α1-subunit immunoreactive clusters (Fig. 2B3; thick arrows), implying GABAergic synapses devoid of α1-GABA_A_Rs, probably containing α2-GABA_A_Rs (see above). Quantification of the density of α1-subunit immunoreactive clusters contacted by VGAT immunopositive puncta, compared to the total density of α1-subunit immunoreactive clusters, revealed that ∼69% of α1-subunit immunoreactive clusters are located within close proximity to putative GABAergic axon terminals (mean ± SEM; total α1-subunit density, 3.5 ± 0.03 clusters *per* 10 μm^2^ *versus* α1 subunit-VGAT density, 2.4 ± 0.04 clusters *per* 10 μm^2^, n = 3 mice).

Given the close overlap of the α1-and α2-subunit immunoreactivity patterns in the core, we examined whether any co-localisation between α1-and α2-subunit immunoreactive clusters was detectable. Numerous co-localised clusters, immunoreactive for both the α1-and α2-subunits were detectable (Fig. 2C1-3). However, quantification of the density of α1-and α2-subunit co-localised clusters (mean ± SEM, 1.1 ± 0.05 clusters *per* 10 μm^2^), compared to the total α1-subunit (mean ± SEM, 3.5 ± 0.05 clusters *per* 10 μm^2^) and α2-subunit (mean ± SEM, 4.8 ± 0.02 clusters *per* 10 μm^2^) immunoreactive clusters revealed that ∼ 31% and ∼23% of total α1-and α2-subunit clusters were co-localised respectively. This analysis implies that some populations of GABAergic synapses of NAc core MSNs are likely to contain receptors incorporating both α1-and α2-subunits and/or synapses containing distinct populations of both α1-and α2-GABA_A_Rs.

### 3.3 The effect of ELA on NAc GABA_A_R subunit mRNA and protein expression in adulthood

To investigate the influence of prior ELA upon GABA_A_R-subunit expression we first employed qPCR to compare the relative subunit mRNA levels in male (>2 months) control and ELA mice. Importantly, in adult mice, prior ELA produced a significant reduction of mRNA expression for the α2-subunit in accumbal tissue “punches” compared to WT controls (p < 0.0001), with no significant effect on the mRNA expression levels for the α1, α4, or δ subunit genes (p > 0.05, Table 2). These data indicate that in the NAc, ELA selectively influences the expression of the α2-subunt mRNA in the adult. Note in common with ELA, we reported that the accumbal mRNA levels of α1-and α4-subunits were similarly not changed in the α2^−/−^ mouse (Dixon et al., 2010).

**Table 2:** ELA selectively reduces accumbal GABRA2 mRNA.

Gene	PCR Reaction Efficiency	Expression	Std. Error	95% C.I	P(H1) = p-value	Result
*Gapdh*	0.956	1.005				
*Ub2q1*	0.952	0.995				
*Gabra1*	0.984	0.948	0.772–1.171	0.632–1.412	0.259	No change
*Gabra2*	1.0	0.645	0.531–0.790	0.423–0.935	< 0.0001	Decreased
*Gabra4*	0.944	0.951	0.789–1.152	0.660–1.371	0.245	No change
*Gabrd*	0.85	1.073	0.856–1.342	0.676–1.643	0.180	No Change

The relative expression of the four GABA_A_R subunit genes investigated (Gabra1, Gabra2, Gabra4, Gabrd) normalised to the two reference genes Gapdh and Ub2q1 in NAc tissue punches of ELS versus control mice. P(H1), as the central outcome of the Pair Wise Fixed Reallocation Randomisation Test^©^ and indicated in the Table, represents the probability of the alternate hypothesis that the difference between the sample and control groups is due only to chance (see Methods Section 2.8). The mean ratio of Gabra2 was 0.645 in the ELA sample group compared to the WT group (1.0) with the true population effect between 0.423 and 0.935 (95% C.I.), which is sufficient to reject the null hypothesis of no difference at the p < 0.05. The applied Pair Wise Fixed Reallocation Randomisation Test^©^ at 10,000 random reallocations of samples and controls further proved the downregulation of Gabra2 to be highly significant (P(H1) = p < 0.0001).

Immunohistochemistry was performed on tissue from male (>2 months) WT control and WT ELA mice to determine whether the ELA-induced alteration in GABA_A_R subunit mRNA expression translated to the protein level. We focussed on the α1-and α2-subunits, which as shown above in the NAc. core form the predominant synaptic GABA_A_R isoforms (Hörtnagl et al., 2013).

**Fig. 3:**
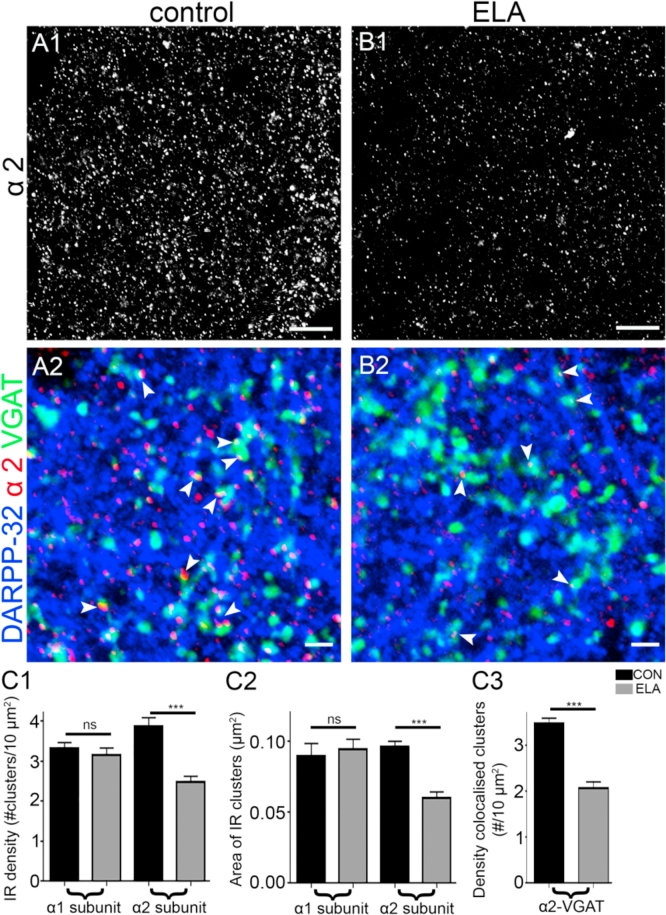
Prior experience of early life adversity produces a selective decrease in GABA_A_R α2-subunit immunoreactivity in the mouse NAc core. **(A1)** A representative image of α2-subunit immunoreactivity in the NAc core of a WT control mouse aged two months. **(A2)** shows a composite image of immunoreactivity for the α2-subunit (red), DARPP-32 (blue) and VGAT (green). The arrowheads highlight the significant degree of association between the α2-subunit and the VGAT signal. **(B1)** Illustrates the comparative levels of α2-subunit immunoreactivity in tissue derived from ELA mice processed and imaged under identical conditions. **(B2)** In comparison to control tissue, the association of the α2-subunit with VGAT immunoreactive clusters (arrowheads) appears dramatically reduced in WT ELA tissue. In addition, the α2-subunit immunoreactive clusters are noticeably smaller compared to those in tissue from WT control mice **(C)** Bar charts (mean ± s.e.m.) illustrating the influence of prior ELA upon **(C1)** the cluster density **(C2)** the cluster area for the α1-and α2-subunit immunoreactivity and **(C3)** the density of VGAT/α2-subunit co-clusters. Note ELA produced a significant decrease in **(C1)** the number, **(C2)** the size of α2-subunit immunoreactive clusters and **(C3**) in the number of VGAT/α2-subunit co-clusters. By comparison prior ELA had no effect on these parameters for the α1-subunit clusters. (***p < 0.001 unpaired Student's t-test; n = 5). ns = non-significant. Scale bars: **(A1, B1)** 10 μm; **(A2, B2)** 3 μm. (For interpretation of the references to colour in this figure legend, the reader is referred to the web version of this article.)

A clear difference in the intensity of immunoreactivity for the α2-subunit was evident in tissue obtained from control compared with ELA mice (Fig. 3A1, B1), processed and imaged under identical conditions. Quantitative analysis of the α2-subunit immunoreactive clusters confirmed a significant decrease, not only of their density (mean ± SEM: control = 3.8 ± 0.14 clusters *per* 10 μm^2^ *versus* ELA, 2.5 ± 0.14 clusters *per* 10 μm^2^; n = 5 mice; t_(8)_ = 5.254, p = 0.0008, unpaired Student's *t*-test), but also in their area (mean ± SEM, control = 0.095 ± 0.004 μm^2^, n = 5 mice *versus* ELA, 0.060 ± 0.004 μm^2^, n = 5 mice; t_(8)_ = 6.345, p = 0.0002, unpaired Student's *t*-test). Notably, there were no significant differences in the density of co-labelling for neuroligin 2, a protein located exclusively within inhibitory synapses (Varoqueaux et al., 2004) confirming that equivalent fields of view and proportions of synapses within control and ELA tissue were selected for comparison (mean ± SEM; control, 5.3 ± 0.2 clusters *per* μm^2^ *versus* ELA, 5.8 ± 0.1 clusters *per* μm^2^, n = 5 animals each; t_8_ = 0.8061, p = 0.43, unpaired Student's *t*-test). In agreement with the decreased density of α2-subunit immunoreactive clusters, there was a significant decrease in the number of clusters contacted by VGAT immunopositive puncta (mean ± SEM; control α2-subunit/VGAT density = 3.5 ± 0.11 clusters *per* 10 μm^2^ *versus* ELA α2-subunit-VGAT density = 2.1 ± 0.13 clusters *per* 10 μm^2^, t_(8)_ = 8.10998, p = 0.0004, unpaired Student's *t*-test, n = 5 mice; Fig. 3A2, B2, C1-3). Collectively, these findings suggest a significant decrease in the expression of the GABA_A_R α2-subunit in MSN synapses in adulthood, as a consequence of ELA.

This effect of ELA appeared to be restricted to the α2-subunit, as there were no significant differences in either the density of α1-subunit immunoreactivity (mean ± SEM; control = 3.3 ± 0.1 clusters *per* μm^2^ *versus* ELA = 3.1 ± 0.2 clusters *per* μm^2^, n = 5 mice each; t_(8)_ = 0.7675, p = 0.4648, unpaired Student's *t*-test), or the area of individual clusters (mean ± SEM; control = 0.09 ± 0.01 μm^2^ *versus* ELA = 0.09 ± 0.02 μm^2^, n = 5 mice each; t_(8)_ = 0.4746, p = 0.6478, unpaired Student's *t*-test) in tissue obtained from control and ELA mice, processed and imaged under identical conditions (Fig. 3C1,2).

**Fig. 4:**
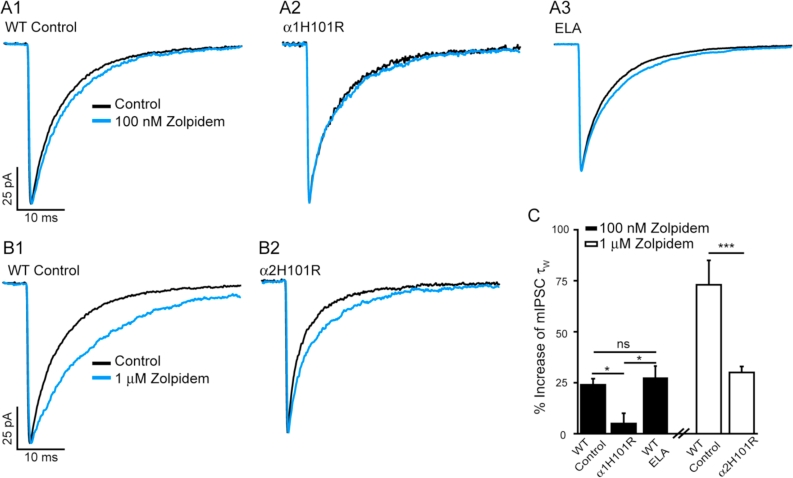
Mouse NAc core MSNs express synaptic α1-GABA_A_ receptors, which are not influenced by prior ELA. **(A1-3, B1-2)** Superimposed, representative averaged traces of GABA_A_R-mediated mIPSCs recorded from NAc core MSNs obtained from wild type (WT) control, α1H101R, α2H101R and ELA mice before (black) and after (blue) the application of either **(A1-3)** 100 nM, or **(B1-2)** 1 μM zolpidem. The amplitude of these events are normalised to the corresponding control for that MSN to aid comparison of the effect of zolpidem on the mIPSC decay. **(C)** A bar chart showing the effect of zolpidem (100 nM & 1 μM) (expressed as % increase; mean ± sem n = 5–11; of paired experiments *per* condition) on the mIPSC decay (τ_W_) of MSNs derived from WT, α1H101R, α2H101R and ELA mice. A low concentration of zolpidem (100 nM) significantly prolonged the mIPSC decay of MSNs derived from WT, and of ELA mice, but not that of α1H101R MSNs. Zolpidem (1 μM), caused a further prolongation of WT mIPSCs. The effect of this greater concentration of zolpidem was reduced, by the α2H101R mutation. Collectively these results demonstrate for WT MSNs the presence of synaptic α1-and α2-GABA_A_Rs and that prior ELA did not influence the enhancing effects of an α1-GABA_A_R selective concentration of zolpidem. The statistical significance (panel **C**) was determined by a one-way RM ANOVA followed by a Tukey *post-hoc* test *p < 0.05, ***p < 0.001, ns = non-significant. (For interpretation of the references to colour in this figure legend, the reader is referred to the web version of this article.)

To complement the immunohistochemistry and establish the presence of functional synaptic α1-and α2-GABA_A_Rs we investigated in paired recordings the actions of zolpidem on mIPSCs. We demonstrated for thalamo-cortical neurons that a low concentration (100 nM) of zolpidem selectively prolonged mIPSCs mediated by synaptic α1-GABA_A_Rs, whereas 1 μM zolpidem additionally influenced α2-and α3-GABA_A_Rs (Peden et al., 2008). Note zolpidem has little effect on α5-GABA_A_Rs (Pritchett and Seeburg, 1990). We therefore compared the action of zolpidem on mIPSCs of WT control mice, with those of mice engineered to express an H101R mutation in either the α1-or the α2-subunit, thereby rendering synaptic receptors incorporating these subunits insensitive to benzodiazepines and to zolpidem (*e.g.* Peden et al., 2008). In agreement with the immunohistochemistry, zolpidem (100 nM) prolonged the mIPSC decay of MSNs derived from WT mice (control τw = 8.5 ± 0.7 ms, zolpidem = 10.7 ± 1.1 ms; n = 8; t_(7)_ = 5.024, p = 0.0015, paired *t*-test), but had no significant effect on mIPSCs recorded from MSNs of the α1H101R mouse (control τw = 7.7 ± 0.4 ms; zolpidem = 8.1 ± 0.6 ms, n = 5, t_(4)_ = 1.133, p = 0.3205, paired *t*-test) –Fig. 4A1,2. To facilitate comparison across these mouse groups the effect of zolpidem was expressed as a percentage of control (Fig. 4C). A one-way RM ANOVA confirmed the effect of zolpidem (100 nM) to be dependent on mouse group (F(2,23) = 4.714, p = 0.02), with a significant difference between the effect of zolpidem upon mIPSC decay recorded from WT and α1H101R mice (p = 0.046, *post hoc* Tukey test). A greater concentration of zolpidem (1 μM), further prolonged the mIPSCs of WT MSNs (control τw = 7.5 ± 0.2 ms, zolpidem τw = 13.3 ± 0.8 ms; n = 8 t_(7)_ = 7.424, p = 0.0001, paired *t*-test) - Fig. 4B1, consistent with additional engagement of α1-GABA_A_Rs and/or the presence of synaptic α2-GABA_A_Rs. Zolpidem (1 μM) also significantly prolonged the mIPSCs recorded from the MSNs of the α2H101R mouse (control τw = 6.3 ± 0.3 ms; zolpidem 1 μM τw = 8.3 ± 0.3 ms, n = 7, t(6) = 8.173, p = 0.0002, paired *t*-test) (Fig. 4B2). Again, to facilitate comparison across these mouse groups the effect of zolpidem (1 μM) was expressed as a percentage of control (Fig. 4C). Statistical analysis revealed a significant influence of the mouse group on the zolpidem (1 μM) effect (F(2,19) = 5.069, p = 0.0172, one-way RM ANOVA), being reduced by the α2H101R mutation compared to WT (p = 0.0002, *post-hoc* Tukey test) - Fig. 4B and C. These results confirm the presence of synaptic α2-GABA_A_Rs in addition to the α1-GABA_A_Rs.

The qPCR and immunohistochemistry data suggested that in contrast to the α2-subunit, the MSN expression of the α1-subunit, was not influenced by ELA. In paired recordings an α1-subunit selective concentration (100 nM) of zolpidem prolonged the mIPSCs of MSNs from WT ELA (control τw = 8.1 ± 0.4 ms; zolpidem τw = 10.2 ± 0.5 ms n = 11, t_(10)_ = 4.998, p = 0.0005, paired *t*-test). To aid a comparison across mouse groups the effect of zolpidem is shown as percentage increase of τw, which is not significantly different between the WT control and WT ELA (p = 0.958, *post-hoc* Tukey test), further suggesting prior ELA to have little, or no impact upon α1-subunit expression (Fig. 4A3, C).

In conclusion, given the immunohistochemistry and electrophysiology findings, the ELA-induced decrease of the mIPSC amplitude is consistent with the reduced number of post-synaptic α2-GABA_A_Rs. Both the ELA and the α2^−/−^ condition additionally decreased the mIPSC frequency. Such an effect may imply that both prior ELA and genetic deletion of the α2-subunit in common decrease either vesicular GABA release, or inhibitory innervation. However, alternatively the altered frequency may reflect a substantial post-synaptic loss of α2-GABA_A_Rs at some inhibitory synapses, such that the remaining receptors are not sufficient to produce detectable phasic events *i.e.* apparently silent synapses. In support, although ELA had no effect on neuroligin expression (a postsynaptic marker of GABA-ergic synapses), the number of VGAT α2-subunit co-clusters was decreased. However, confirmation of this hypothesis would require ultra-structure analyses, using immunohistochemistry at the transmission electron microscope level.

### 3.4 ELA, in common with the deletion of the α2-GABA_A_R subunit, influences the acute locomotor effects of cocaine and sensitisation

To investigate whether ELA influences the behavioral effects of cocaine we compared the ability of this stimulant to acutely increase locomotion and to cause locomotor-sensitisation upon repeated daily cocaine administration in WT control and WT ELA. Given the ELA-induced impairment of α2-GABA_A_R subunit expression, their behavioral response to cocaine was additionally compared to that of the α2^−/−^ mouse. However, prior to this study we determined the behaviour of the α2^−/−^ dam in the control cage during p 2–9 of the life of the pups. In comparison to WT control dams, the α2^−/−^ mothers presented with significantly fewer sorties from the nest (WT control = 29.6 ± 3.03/hr. n = 9; α2^−/−^ = 13.9 ± 2.5/hr. n = 6; t_(13)_ = 3.67; p = 0.0028), perhaps reflecting a “freezing” behaviour associated with the anxiogenic phenotype of α2^−/−^ mice (Dixon et al., 2008, Koester et al., 2013). By comparison, WT dams undergoing ELA exhibit an increased number sorties, compared with WT controls. These contrasting behaviours of a global knockout with a partial knockdown during neonatal development warrant further studies to 1) compare ELA and α2^−/−^ mice in tests predictive of anxiogenic behaviour, and 2) determine the impact of ELA upon α2-subunit expression in other brain regions. In this respect, the influence of α2-subunit on the anxiolytic actions of diazepam in particular hippocampal neuronal populations is pertinent (Engin et al., 2018).

**Fig. 5:**
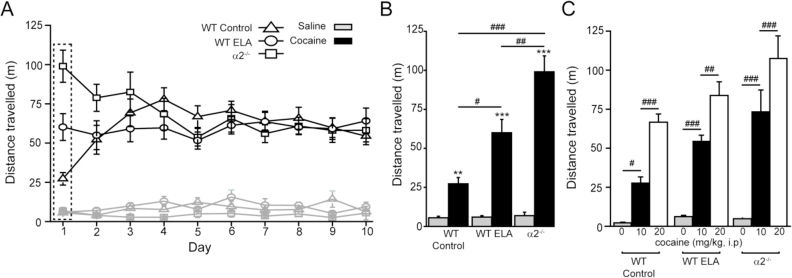
A comparison of the effects of prior ELA and the genetic deletion of the α2-GABA_A_R subunit, on the acute locomotor effects of cocaine and sensitisation. **(A).** A line graph illustrating the locomotor response (distance travelled in metres [m] during the first 15 min), to a single daily *i.p* injection of either 10 mg/kg cocaine (black), or saline (grey) for WT control (triangle, saline n = 9, cocaine n = 19), WT ELA (circle, saline n = 9, cocaine n = 14) and α2−/− mice (square, saline n = 12, cocaine n = 25), plotted over the 10 test days (session by mouse group by treatment interaction: F_(18, 738)_ = 1.729, p = 0.03 two way RM ANOVA). **(B).** A histogram illustrating the acute day 1 response (see also the grey dashed box in panel **A**) to cocaine (black) and saline (grey) for WT control (drug versus saline t_(26)_ = 3.665, p = 0.0011), WT ELA (drug versus saline t_(24)_ = 5.833, p = 0.0001)and α2^−/−^ mice (drug versus saline t_(32)_ = 5.321, p = 0.0001). **(C).** A histogram showing the locomotor response to an *i.p* injection of either saline (grey n = 11 *per* mouse group), 10 mg/kg cocaine (black n = 11 *per* mouse group), or 20 mg/kg cocaine (white n = 11 *per* mouse group). In agreement with Panel **B,** again a single *i.p.* injection of 10 mg/kg cocaine produced a significant increase in the locomotor activity of WT control (p = 0.0028), of WT ELA (p = 0.0001) and of α2^−/−^ mice (p = 0.0001) mice compared to saline (*post hoc* Tukey test). Additionally, in drug naïve mice, a higher dose of cocaine (20 mg/kg), further increased the locomotor activity of WT control (p = 0.0007), WT ELA (p = 0.0053) and of α2^−/−^ mice (p = 0.0001) mice *versus* the 10 mg/kg dose, **p < 0.01, ***p < 0.001 unpaired Students t-test, #p < 0.05, ##p < 0.01, ###p < 0.001 *post hoc* Tukey test.

A daily administration protocol, repeated for 10 days, altered locomotor activity (during a 15 min test period, see Methods) in a manner dependent on mouse group, treatment and day (session by mouse group by treatment interaction: F_(18, 738)_ = 1.729, p = 0.03 two way RM ANOVA; Fig. 5A). A *post-hoc* analysis, revealed that on the first test day, the acute administration of cocaine (10 mg/kg *i.p.*) produced a significant increase in locomotion for WT control mice compared to the respective saline locomotor response (saline = 5.5 ± 1.0 m; cocaine = 27.3 ± 4.0 m; t_(26)_ = 3.665, p = 0.0011), for WT ELA (saline = 6.0 ± 0.9 m; cocaine = 60.0 ± 8.5 m; t_(24)_ = 5.833, p = 0.0001) and for α2^−/−^ mice (saline = 6.9 ± 2.2 m; cocaine = 99.0 ± 10.3 m; t_(32)_ = 5.321, p = 0.0001; Fig. 5A and B). In comparison to WT control mice, this acute locomotor response to cocaine was significantly greater for both the WT ELA (p = 0.0228 *post hoc* Tukey) and for the α2^−/−^ mice (p = 0.0001 *post hoc* Tukey) - Fig. 5A and B.

Repeated drug, but not saline administration (saline; session x mouse group effect F_18, 243)_ = 1.242, p = 0.2286 two way ANOVA), resulted in a significant influence on the locomotor effect of cocaine (cocaine; session by mouse group effect F_(18, 495)_ = 4.395, p = 0.0001 one way ANOVA). Analysis of individual mouse groups revealed a significant sensitisation for WT control upon repeated administration (F_(9, 189)_ = 6.105, p = 0.0001 one way ANOVA), but not for the WT ELA (F_(9, 139)_ = 0.2787, p = 0.9794 one way ANOVA). For the α2^−/−^ mouse group inspection of Fig. 5A revealed that the enhanced acute response to cocaine on day 1 decreased upon subsequent repeated daily administration (F_(9, 249)_ = 4.414, p = 0.0001 one way ANOVA). Note by day 3 all mouse groups (WT control, WT ELA and α2^−/−^) exhibited a similar effect to cocaine (F_(2, 57)_ = 1.050, p = 0.357 one way ANOVA), which was maintained until the last day of testing (Fig. 5A).

The lack of sensitisation to cocaine, observed in WT ELA and α2^−/−^ mice, may be an indirect consequence of the enhanced acute response to cocaine *i.e.* the result of a “ceiling effect”. We therefore compared the acute cocaine dose-response relationship for WT control, WT ELA and α2^−/−^ mice (0, 10, 20 mg/kg cocaine) in an additional cohort of mice (see Methods and Fig. 5C). The acute locomotor response was sensitive to the cocaine dose and as before also to the mouse group (dose x mouse group interaction; F_(4,90)_ = 3.23, p = 0.0159; mouse group F_(2,90)_ = 14.37, p = 0.0001; dose F_(2,90)_ = 115.5, p = 0.0001 two way ANOVA; Fig. 5B and C). Importantly, in previously drug naïve mice, the locomotor stimulant action of 20 mg/kg cocaine was significantly more effective than the 10 mg/kg dose for all mouse groups (WT control mice p = 0.0007, WT ELA mice p = 0.0053, α2^−/−^ mice p = 0.0001 Tukey *post hoc* analysis; Fig. 5C).

In summary, in comparison to control WT mice, the ELA WT and the α2^−/−^ mice exhibit an increased acute response to cocaine, but blunted sensitisation upon repeated daily administration. These behavioral similarities to cocaine suggest an involvement of the GABA_A_R α2-subunit in the altered behaviour of the ELA mouse. However, the causative factors of the limited nesting model that result in the modified cocaine behaviour and accumbal α2-GABA_A_R subunit expression/function are not known. Possibilities include – an increase in the number of sorties from the nest, the influence of an “anxious” mother upon the developing pups, the quality of the mother's milk, changes to suckling behaviour and nest temperature, or more likely some combination of these factors (Walker et al., 2017). Clearly, the differential effect upon the sortie behaviour of the ELA and the α2^−/−^ dams suggests that this particular aspect of the disruption of the mother-pup interaction alone is not the prime driver of the behavioral changes.

## 4 Discussion

Clinical and animal studies provide compelling evidence to associate experience of stressful events during early development and a subsequent increased incidence of psychiatric disorders, including depression, anxiety and substance misuse (Lupien et al., 2009, Teicher et al., 2016, Syed and Nemeroff, 2017). We employed an established naturalistic mouse model of ELA, which provides face and construct validity and reproducibility (Rice et al., 2008, Baram et al., 2012, Gunn et al., 2013, Walker et al., 2017). In adult mice prior ELA caused a selective decrease of α2-subunit expression in NAc core MSNs, thereby impairing phasic inhibition. Such mice exhibited a dysregulated locomotor response to acute and chronic cocaine, a profile similar to the α2^−/−^ mouse.

### 4.1 ELA selectively influences phasic inhibition and NAc α2-GABA_A_R expression

Given the role of the NAc in drug reward and abuse, we investigated the impact of ELA on accumbal GABA-ergic inhibition and α2-subunit expression. The adult NAc core exhibited much greater α2-subunit immunoreactivity than the shell, which mainly co-localised with VGAT staining, implying a synaptic locus. ELA decreased MSN mIPSC amplitude and frequency, a profile similar to the adult α2^−/−^ mouse. Importantly, electrophysiological, immunohistochemical and PCR analysis revealed a selective reduction of α2-subunit, but not α1-subunit expression to cause this deficit of synaptic inhibition.

Accumbal core MSNs additionally express extrasynaptic GABA_A_Rs composed of α4-, β- and δ-subunits, that mediate a tonic current (Maguire et al., 2014). ELA had no effect on α4-, or δ-mRNA expression, or on the tonic current. During early neonatal development, α2-subunit expression is much greater than α1-subunit expression (Laurie et al., 1992, Fritschy et al., 1994). Similarly, δ-subunit expression is developmentally delayed. Whether this chronology of subunit expression influences the selective effects of ELA upon GABA_A_R-subunit expression is not known.

We focused on the NAc core, given the relevance to behavioral sensitisation caused by psychostimulants (Pierce et al., 1996, Cadoni and Di Chiara, 1999, Ito et al., 2000) and clinical studies demonstrating an influence of the *Gabra2* haplotype on accumbal activation during reward anticipation (Heitzeg et al., 2014). However, changes to GABA_A_R-subunit expression in other brain regions are apparent in related models of neonatal stress. Interestingly, α2-subunit expression is increased in the amygdala and prefrontal cortex of ELA-exposed adult rats (Gondre-Lewis et al., 2016). Furthermore, rodents experiencing different levels of maternal care, exhibit region-selective expression changes in a variety of GABA_A_R subunits (Caldji et al., 2003, Caldji et al., 2004). Exposure to chronic stress, or to chronic corticosterone, produced region-selective changes of α2-subunit expression for high *vs* low anxiety rats (Wisłowska-Stanek et al., 2013, Skórzewska et al., 2014). The stress-induced plasticity of the α2-subunit is intriguing, as the anxiolytic effects of barbiturates and benzodiazepines are mediated by α2-GABA_A_Rs and α2^−/−^ mice exhibit an anxiogenic phenotype (Low et al., 2000, Morris et al., 2006, Dixon et al., 2008, Atack, 2011, Smith et al., 2012). Studies determining the influence of ELA on α2-subunit expression in other brain regions are warranted *e.g.* the hippocampus where α2-GABA_A_Rs expressed in particular neurons appear crucial in mediating diazepam-induced anxiolysis (Engin et al., 2018). Furthermore, as stress paradigms in adult rodents influence GABA_A_R-subunit plasticity (Mody and Maguire, 2012) it would be of interest to determine if accumbal α2-subunit expression was similarly selectively impaired in mature mice following stress.

### 4.2 ELA influences cocaine behaviour: a role for α2-GABA_A_Rs?

In adult WT mice, ELA greatly increased the locomotor response to acute cocaine and blunted behavioral sensitisation to repeated administration, findings consistent with previous reports (Moffett et al., 2007). Alternative rodent models of ELA, employing protracted maternal separation, or neonatal isolation, also increased locomotor activity to acute cocaine and/or blunted sensitisation to repetitive dosing (Li et al., 2003, Kikusui et al., 2005, Gracia-Rubio et al., 2016). Although the human correlate of sensitisation is not clear, in rodents it provides a paradigm to investigate how stress, or drugs of abuse, induce enduring behavioral and neuronal plasticity (Yap and Miczek, 2008). Here, the cocaine phenotype (enhanced acute effect and impaired sensitisation) of ELA mice was similar to that of mice raised under control conditions, but lacking the α2-subunit gene (α2^−/−^), suggesting that α2-subunit plasticity may contribute to the influence of neonatal stress upon the effects of cocaine in adults. The down-regulation of α2-GABA_A_Rs in the NAc core may be particularly pertinent to psychostimulant-mediated behaviors. WT but not α2^−/−^ mice exhibit reduced drug intake in the first few days of self-administration, supporting a protective role of α2-GABA_A_Rs against drug abuse (Dixon et al., 2014). Furthermore, in rat accumbens a transient increase in α2-GABA_A_R subunit surface expression accompanies withdrawal from cocaine-self administration, with no changes to α1-or α4-subunits (Purgianto et al., 2016).

### 4.3 Stress and cocaine cross-sensitisation: dopaminergic and glutamatergic plasticity and a putative role for α2-GABA_A_Rs

Behavioral sensitisation from chronic psychostimulants is mirrored by exposure to acute, or chronic stress (Lu et al., 2003, Yap and Miczek, 2008, Garcia-Keller et al., 2013). Although our primary focus concerned the influence of ELA upon GABA-ergic transmission, plasticity of glutamatergic and dopaminergic systems plays an important role in the neuroadaptations underlying the facilitation by acute stress of the locomotor and rewarding effects of psychostimulants, including cocaine (Saal et al., 2003, Pacchioni et al., 2007, Kalivas, 2009). In mesolimbic circuitry AMPA receptor (AMPAR) expression increased both in response to cocaine (Churchill et al., 1999, Boudreau and Wolf, 2005, Wolf and Ferrario, 2010) and to stress (Pierce et al., 1996, Garcia-Keller et al., 2013). We reported that prior ELA increased both phasic and tonic excitation of hypothalamic parvocellular CRF neurons (Gunn et al., 2013). However, here ELA had no effect on phasic and tonic excitation (mediated by synaptic AMPARs and extrasynaptic NMDARs respectively) of NAc core MSNs, although our recordings from MSN somata do not exclude changes to glutamatergic function occurring in other cellular domains (dendrites).

We have not explored the impact of ELA upon accumbal dopaminergic transmission. However, our PVN studies suggest ELA to increase CRF release (Gunn et al., 2013). As the NAc expresses CRF receptors that directly influence dopamine release, an effect influenced by prior stress (Lemos et al., 2012), it is conceivable that an ELA-induced increase of CRF levels, either circulating, or emanating from direct efferent projections to the NAc (Zhang et al., 2016) contributes to the ELA behavioral phenotype and accumbal receptor plasticity. Additionally, α2-GABA_A_Rs can influence cocaine behavioral sensitisation in the NAc *via* mechanisms downstream and independent from changes to dopaminergic transmission (Morris et al., 2008, Dixon et al., 2010). We speculate that a partial loss of α2-GABA_A_Rs in the NAc may, under certain conditions, *e.g.* stress exposure, play an important role in skewing the output of motivational circuits towards addictive behaviors. Neuronal modelling studies (Wolf et al., 2005, Moyer et al., 2014) suggest that this effect may be mediated by a loss of feed-forward inhibition in the MSN soma and lateral inhibition amongst MSNs, thereby altering the gain of the striatal network, thus biasing the output of MSN ensembles to favour “addictive” behaviour (Stephens et al., 2017).

### 4.4 ELA, α2-GABA_A_Rs and cocaine-mediated behaviors: clinical relevance

Our findings complement clinical studies linking haplotypes and individual SNPs of the *Gabra2* gene with susceptibility to several substances of abuse, including cocaine (Covault et al., 2004, Edenberg et al., 2004, Enoch, 2011, Stephens et al., 2017). For cocaine, the association for genetic variations only becomes significant when accompanied by early trauma experience (Enoch et al., 2010, Enoch, 2011). The SNP rs299858 identifies a risk haplotype, common across multiple studies of addicted populations, including individuals abusing cocaine (Covault et al., 2004, Lappalainen et al., 2005, Fehr et al., 2006, Enoch et al., 2010). Importantly, an iPSC culture model reported reduced mRNA levels for the rs299858 harbouring *Gabra2* gene (Lieberman et al., 2015). Supporting an accumbal locus, adolescents harbouring the *Gabra2* (rs279858) haplotype exhibit greater NAc activation during reward anticipation (Heitzeg et al., 2014). Therefore, collectively it is conceivable that genetic- and stress-impaired accumbal α2-GABA_A_R expression may synergise, providing a mechanism whereby early trauma influences genetic susceptibility to substance abuse.

## 5 Conclusion

In preliminary studies we found ELA decreased accumbal α2-subunit expression by P20, revealing this perturbation to be well-maintained from neonate to adult. Indeed, exposure to stressful events during sensitive developmental periods may produce long-lasting changes in the plasticity of mesolimbic neurocircuitry (Peña et al., 2017). Future investigation may elucidate the molecular mechanisms (*e.g.* epigenetics) underpinning such enduring changes to α2-subunit expression. Given that female rodents appear more resilient to ELA, an investigation of gender influence is warranted (Walker et al., 2017). Additionally, we require a better understanding of the role of α2-GABA_A_Rs to influence MSN excitability *e.g.* their impact upon distinct glutamatergic inputs to the NAc implicated in stress-resilience and stress-susceptibility (Bagot et al., 2015, Christoffel et al., 2015). Although recognising that ELA causes plasticity in other brain regions, the development of selective positive allosteric modulators of α2/3-GABA_A_Rs (Atack, 2011) may be useful in treating individuals at risk for substance abuse. Finally, these findings may lead to a better understanding of the clinical literature, which associates early-life adversity and α2-GABA_A_R subunit haplotypes with substance abuse.

## Conflicts of interest statement

This is to state that the authors do not have any conflicts of interest.

## Author contributions

J.J.L., D.B., J.D.S., D.N.S., U.Z. designed research; S.J.M., E.P.M., J.D.S., L.C., M.L. B.G.G. performed the research; S.J.M., E.P.M., U.Z., M.L., D.B., J.D.S., L.C. analysed the data. J.J.L., D.B., J.D.S., D.N.S., S.L.K. C.I.D. wrote the paper.
